# The Impact of Weather on Women’s Tendency to Wear Red or Pink when at High Risk for Conception

**DOI:** 10.1371/journal.pone.0088852

**Published:** 2014-02-21

**Authors:** Jessica L. Tracy, Alec T. Beall

**Affiliations:** Department of Psychology, University of British Columbia, Vancouver, British Columbia, Canada; University of Goettingen, Germany

## Abstract

Women are particularly motivated to enhance their sexual attractiveness during their most fertile period, and men perceive shades of red, when associated with women, as sexually attractive. Building on this research, we recently found that women are more likely to wear reddish clothing when at peak fertility (Beall & Tracy, 2013), presumably as a way of increasing their attractiveness. Here, we first report results from a methodological replication, conducted during warmer weather, which produced a null effect. Investigating this discrepancy, we considered the impact of a potentially relevant contextual difference between previous research and the replication: current weather. If the red-dress effect is driven by a desire to increase one’s sexual appeal, then it should emerge most reliably when peak-fertility women have few alternative options for accomplishing this goal (e.g., wearing minimal clothing). Results from re-analyses of our previously collected data and a new experiment support this account, by demonstrating that the link between fertility and red/pink dress emerges robustly in cold, but not warm, weather. Together, these findings suggest that the previously documented red-dress effect is moderated by current climate concerns, and provide further evidence that under certain circumstances red/pink dress is reliably associated with female fertility.

## Introduction

Sexual intercourse typically results in conception only within the few days of a woman’s cycle prior to and during ovulation [1], making it potentially adaptive for women to advertise their fertile window in an observable manner that attracts male attention. Indeed, females of many closely related primate species experience highly visible physical changes during ovulation (e.g., chimpanzees, bonobos), which have the effect of increasing male attention and sexual interest [2]–[4] (see [5]; but also see [6]). Human women, of course, do not show any visually observable physical changes associated with ovulation, and until recently, scientists also had not found any clearly observable, behavioral display linked to human ovulation. As a result, some researchers have concluded that it may be most adaptive for women to hide their fertile window from men, so as to promote male investment across the cycle [7].

However, other studies have found that women show psychological changes during this period; in particular, they become highly motivated to enhance their attractiveness [8]. Indeed, a selective tendency to engage in behaviors that increase one’s attractiveness during the fertile window may be the most adaptive female response to the female ovulatory cycle, given that such behaviors might increase the pool of viable mates a woman has to choose from during the time when conception is most likely, while also preventing men from obtaining concrete knowledge about the precise time when mating is most likely to pay off. Building on this account, as well as evidence suggesting that men perceive shades of red–when associated with women–as sexually attractive [9], we recently examined whether displays of red might be used by women to increase their attractiveness during this period. Specifically, we tested whether women preferentially dress in reddish colored clothing during peak fertility [10].

### The Attractiveness of Red

Across a wide range of cultures, the color red is cognitively associated with love and passion [11], and studies using a range of methods and populations have demonstrated that women’s use of red is linked to sex and romance [12], [13]. For example, one study found that women report a preference for wearing red clothing when imagining situations in which they might meet a potential mate. Based on this finding, the authors concluded that women might use red-colored clothing as a sexual signal to attract potential mates [14]. Other studies have found that men judge women wearing or surrounded by red to be particularly attractive and sexually desirable [9]. This last effect has been demonstrated in both Western industrialized cultures and among members of a non-Western, highly isolated, traditional small-scale society where red carries divergent (and negative) cultural associations, suggesting that the male tendency to find reddish coloration sexually attractive may be a human universal [15].

Several possible explanations have been proffered for men’s attraction to redness on or surrounding women (see [9]); for example, red’s attention-grabbing perceptual properties; the fact that reddish skin tone often indicates sexual arousal in women; and the finding that males of other species show a similar attraction [2], suggesting that these cognitions might have originally emerged in a shared ancestor and be retained in humans. Regardless of the mechanism underlying this effect, the well-documented presence of this tendency among men leads to an interesting prediction regarding women. Given women’s brief fertile window, it may be adaptive for them to dress in a way that increases their sexual attractiveness to men during this period. In light of the evidence for men’s attraction to reddish colors, then, we predicted that women would opportunistically self-adorn in red or pink during times of peak fertility, as part of a broader drive to appear more sexually attractive during this time of the cycle [5], [16]. Alternatively, if the hormones associated with ovulation make women feel sexier during peak fertility [5], women might be inclined to wear clothing with attention-grabbing properties (e.g., red-colored) during this time, even in the absence of any desire to attract men.

### Female Fertility Signaling

A considerable body of prior research suggests that the advertisement of human female fertility is largely hidden, involving vocal, olfactory, and subtle visual changes, but few overt behavioral displays [17], [18]. For example, although two studies suggest that women’s faces are judged as more attractive during peak fertility, the women in these studies posed neutral expressions and removed all cosmetics prior to being photographed, making it unlikely that their increased attractiveness was due to any objectively measureable behavioral display [19], [20].

Other studies have documented fertility-linked psychological changes, particularly in women’s mate-seeking desires and behavioral tendencies: a self-reported increased desire to have sex with men (e.g., [5]), an increased attraction to physical markers of health and masculinity [21], an increased desire to wear revealing clothing [16], and a tendency to wear clothing that leads women to be judged as “trying to look more attractive” [8]. One study found that women at peak fertility actually wore more revealing clothing, but this effect emerged only among partnered women (whose partners were absent) attending Austrian discotheques where, presumably, dressing provocatively does not violate social norms [22]. These caveats are important because, despite several attempts, most of the prior research addressing this question failed to document an objectively observable change in women’s everyday behavior or dress that is linked to ovulation. For example, although Haselton and colleagues [8] found that partnered women were rated as dressing “more fashionably” at peak fertility, this effect was restricted to partnered women. Likewise, although Durante and colleagues [16], [23] found that women at peak fertility reported a desire to purchase and wear sexier clothing when imagining attending a social gathering where they might meet men, no difference emerged in the observed sexiness of the clothing the women actually wore. Consistent with the findings from the Austrian discotheque, Durante and colleagues [16] argued that experimental studies may fail to document an ovulation-linked change in women’s dress because dressing in provocative clothing is often not socially acceptable, particularly for women participating in research on a university campus. As a result, it remains unclear whether there is any salient, observable behavioral display reliably associated with female ovulation.

### Red Dress and Conception Risk

Building on the evidence reviewed above suggesting that women may seek to increase their attractiveness by self-adorning in reddish colors, and should be particularly motivated to do so during peak fertility, we tested whether women are more likely to wear red- or pink-colored clothing during this period, compared to other phases of their menstrual cycle [10]. Results from two independent samples supported this hypothesis. Specifically, we recruited women between the ages of 18–40 from a university campus (*n*  = 24) and via Amazon Mechanical Turk (*n*  = 100) to participate in an online study in which they reported the first day of their last period of menses, which we used to calculate their current conception risk, and the color of the shirt they were currently wearing. In both samples, women determined to be at high conception risk were substantially more likely to report wearing a red or pink shirt compared to women at low risk, 40% vs. 7%, and 26% vs. 8%, with odds ratios of 8.67 and 3.85 (see [10]). These strong statistically significant effects, replicated across two samples, suggest that women at peak fertility show a tendency to dress in reddish clothing, perhaps as a way of increasing their sexual attractiveness to men.

This finding has important implications for our understanding of women’s fertility and ovulation- related behavior. Most notably, it supports the expectation that displays of red and pink may be a fertility cue in women, and indicates that there is a visually salient, publicly observable objective behavior that is associated with female ovulation. However, the underlying mechanism accounting for this association remains unknown. This mechanism may be best explained as a byproduct of other psychological and motivational changes that occur with ovulation. Past research has suggested that women desire to dress in a more sexually appealing way during ovulation; however, studies have largely failed to demonstrate any consistent behavioral change in the sexiness of women’s dress across periods of conception risk [8], [16], [5], [22]. The finding that women at peak fertility are more likely to self-adorn in reddish clothing offers a possible explanation for this discrepancy: Although women at high conception risk may refrain from dressing more provocatively out of social-normative concerns [16], they may nonetheless seek to increase their apparent sexiness by wearing colors known to increase their attractiveness to men, which, at least in North American contexts, are not associated with any social stigma.

If this is the case, then it is likely that the previously documented red-dress effect will not generalize across all contexts, even within North American culture. That is, dressing in red or pink may be a particularly effective means of increasing one’s sexual attractiveness when other options for doing so are limited by social constraints (e.g., on a university campus where it is not socially acceptable for women to wear highly revealing clothing; see [16]). However, in contexts where other means of increasing one’s sexual appeal are readily available (e.g., at a beach, where revealing swimsuits are normative), women at high-conception risk may have less need to rely on reddish adornment as a way of increasing their attractiveness, making the effect less robust. In fact, in such circumstances wearing reddish clothing may feel too extreme, or blatant; women wearing minimal clothing may be less inclined to ensure that those clothing are red, even when at peak fertility, because doing so might make them feel like they are sending too strong a message about their sexual receptivity. Indeed, Durante and colleagues [16] argued that ovulation-linked changes in women’s dress (e.g., wearing provocative clothing) may shift according to fluctuating norms. This raises the possibility that under certain circumstances, such as situations in which it is socially appropriate for women to dress in revealing clothing, the ovulation-linked tendency for women to wear red or pink may be less pronounced or even nonexistent.

To examine this issue, we first sought to replicate our prior findings demonstrating the red dress effect in a new sample of women, and at a different time of year. Our prior research documenting the effect was conducted during the winter (January-April, 2012), a relatively cold period in North America, making it both socially unacceptable and physically uncomfortable for women to dress in revealing or minimal clothing as a way of increasing their sexual appeal. We thus sought to replicate the effect in a time of warmer weather, to examine the extent to which it generalizes across contexts where women have more alternative options for increasing their sexual appeal in a socially acceptable manner.

## Preliminary Replication Study

### Methods

Research methods were approved by the University of British Columbia Behavioral Research Ethics Board; informed consent was written. One-hundred sixteen regularly ovulating women were recruited through Amazon’s Mechanical Turk. To increase our likelihood of accurately assessing participants’ current risk of conception, we requested that women not participate if they were: over forty-years-old, users of hormonal birth control, cigarette smokers, pregnant, or not experiencing regular menstrual cycles (see [24]).

Participants completed an online survey in which they were asked, “What color is the shirt/top you are currently wearing? (If your shirt/top is multicolored, please select the color which is most prevalent).” They were given the following response options: Black, Blue, Brown, Gray, Green, Orange, Pink, Purple, Red, White, Yellow, and “Other.” Next, they were asked, “How many days has it been since the onset of your last period of menses?” They were shown a calendar of the current and past months to help jog their memory and facilitate their counting. Participants’ responses to this question were used to divide them into high (days 6–14; *n*  = 64) and low (day 5 and 15–23, *n*  = 52) conception-risk groups, based on a standard 28-day model of the menstrual cycle [21]. We excluded women whose first day of menses occurred more than 28 days previous to avoid including women with atypical cycles, and women who were within 5 days of the onset of menses (days 0–4; 24–28) to ensure that observed effects were not attributable to menstrual or premenstrual symptoms (see [24]), [25]. In our prior research [10] we had requested that women within 5 days of menses onset not participate. Some did nonetheless, and results were generally similar regardless of whether these women were or were not included in analyses (see [10] for more information).

To address concerns regarding the assessment of menstrual history via self-report [26] (but see [27]), and following our prior research [10], participants were also asked, “Within how many days are you 100% confident in your above estimate?”; they responded using a scale where 1 indicated “0 days (I’m 100% confident)”, 2 indicated “1 day”, 3 indicated “2 days”, 4 indicated “3 days”, 5 indicated “4 days”, 6 indicated “5 days”, and 7 indicated “More than 5 days (I’m not very confident)”. We excluded all participants who responded with 7 (*n*  = 3), and all for whom we could not determine conception-risk category membership with 100% certainty (*n*  = 26). Specifically, we excluded, for example, any participant who indicated that her last menses began 12 days ago but was 100% confident of that estimate within 3 days. In this case, we would assume that her last period began 9–15 days ago, and thus could *not* be included in either the high-risk (days 6–14) or low-risk (days 0–5 and 15–28) group. In contrast, any participant who indicated that her last menses began 10 days ago and was 100% confident within 3 days would be included, because we could assume that her period began 7–13 days ago, placing her firmly within the high-risk group (days 6–14).

### Results and Discussion

Women were classified as wearing a red/pink shirt (*n  = *13) or an other-colored shirt (*n*  = 103). Unexpectedly, women at high-conception risk were no more likely to wear red/pink shirts than women at low risk; 11% vs. 12%, (1, *N*  = 116)  = 0.01, *p*  = .92 (Odds ratio  = 0.94). Conception risk also had no effect on the prevalence of any other shirt color:(1, *N*  = 116)  = 0.29, *p*  = .59 (Odds ratio  = 0.77), for black; (1, *N*  = 116)  = 0.56, *p*  = .45 (Odds ratio  = 0.69), for blue; (1, *N*  = 116)  = 0.34, *p*  = .56 (Odds ratio  = 1.67), for brown;(1, *N*  = 116)  = 0.02, *p*  = .88 (Odds ratio  = 0.92), for gray; (1, *N*  = 116)  = 1.22, *p*  = .27 (Odds ratio  = 0.39), for green; (1, *N*  = 116)  = .17, *p*  = .67 (Odds ratio  = 1.65), for orange; (1, *N*  = 116)  = 1.17, *p*  = .28 (Odds ratio  = 1.96), for purple;(1, *N*  = 116)  = .24, *p*  = .62 (Odds ratio  = 1.34), for white; (1, *N*  = 116)  = 3.37, *p*  = .07 (Odds ratio could not be calculated), for yellow; and(1, *N*  = 116)  = 2.51, *p*  = .11 (Odds ratio could not be calculated), for “other”.

One possible explanation for this discrepancy from our prior research is the difference in time of year when the studies were conducted, and associated change in climate. If, as we suspect, women’s tendency to wear red or pink when at high-conception risk is driven by an enhanced desire to increase their sexual attractiveness during this period, then this tendency may be less pronounced when there are more alternative means of overtly displaying one’s sexual appeal. In warmer weather, women can, without violating social norms, wear revealing or minimal clothing to advertise their sexual appeal, thus potentially obviating the need to wear red or pink. In colder weather, these behaviors are both non-normative and physically uncomfortable. Thus, in cold weather, wearing red or pink clothing may provide a more viable means of increasing one’s sexual attractiveness. As a preliminary investigation of this possibility, we next re-analyzed our prior data [10], separately for the colder and warmer months of data collection.

Specifically, we combined data from two previously collected samples of women (total *N*  = 124), and computed a median split based on data-collection time (*median* date = March 31, 2012). We then examined the effect of conception risk on shirt color within each sub-sample. During the colder months of February-March, conception risk strongly predicted color choice, with 30% of high-risk women wearing red or pink compared to 5% of low-risk women, (1, *N*  = 70)  = 7.61, *p*  = .006 (Odds ratio  = 7.61). However, this effect was reduced to non-significance during April, 25% vs. 12%, (1, *N*  = 54)  = 1.62, *p*  = .20 (Odds ratio  = 2.56).

This pattern is consistent with the hypothesis that women’s tendency to dress in red or pink at peak fertility is the result of women invoking a particularly viable means for increasing their sexual attractiveness during colder weather, when few alternatives for doing so exist. However, given that these analyses were conducted in a *post-hoc* fashion, we next conducted a new experiment to directly test this prediction.

## Experiment

### Methods

Research methods were approved by the University of British Columbia Behavioral Research Ethics Board; informed consent was written. Procedures were the same as in the Preliminary Study, except that data were collected on one of two targeted days that fell within the same season (Winter 2012–13) but varied substantially in temperature. Specifically, we closely observed weather patterns beginning in late Fall 2012. On an unseasonably warm day of winter, December 3rd, we posted our study on Mechanical Turk and recruited a large sample (*n*  = 101) of participants within that day. We then posted the study again on an unseasonably cold day later that winter, January 18 (for this session, two days were required to reach a comparable cell size, *n*  = 108). We chose to compare behavior between two days within the same season (Winter) rather than two days in different seasons (e.g., Winter vs. Summer) in order to minimize the impact of other confounding factors that might vary between seasons, other than temperature (e.g., whether participants are currently attending school).

### Results and Discussion

To verify our quasi-manipulation of weather, participants rated the perceived temperature and warmth of the day they participated in the study, on scales ranging from 1 (“0–9°F”) to 9 (“80–89°F”) and 1 (“extremely cold”) to 8 (“very warm”). The two days differed in both perceived temperature, *F* (208)  = 60.30, *Ms*  = 4.68 vs. 6.23, *p*<.001, and perceived warmth, *F* (208)  = 79.69, *M*s  = 3.18 vs. 4.88, *p*<.001. Using the Farmer’s Almanac, we confirmed that these perceptions were accurate, based on a comparison of *mean* temperatures of each state in which participants resided, with *M*s weighted by participant (*M*s  = 38.76°F for the Cold Days vs. 56.83°F for the Warm Day, *t* (207)  = 14.92, *p*<.001.).

**Figure 1:**
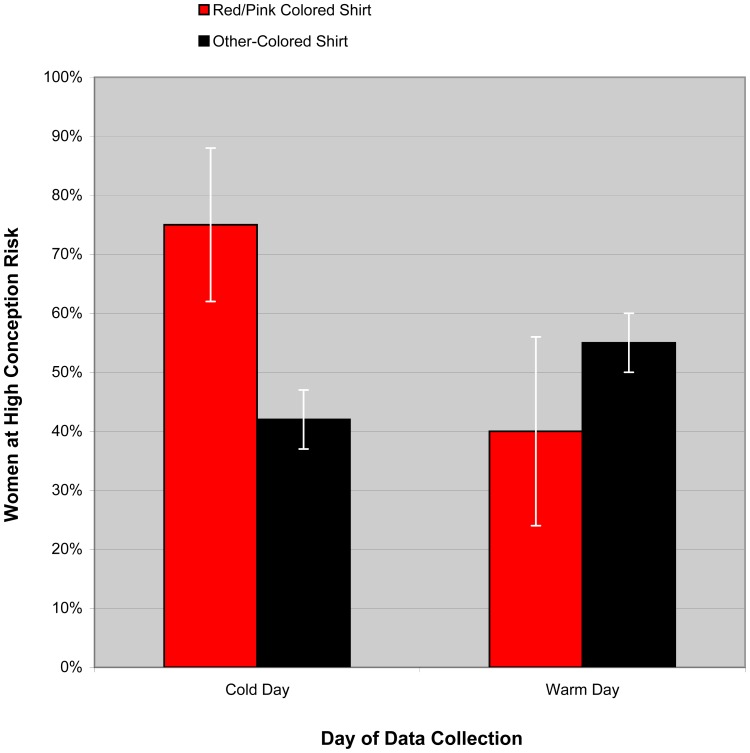
Percentage of women at high-conception risk as a function of shirt color and day of data collection. A significant interaction between day of collection and shirt color, *B*  = 2.06, Wald (1)  = 4.49, *p*  = .03, indicated that, while on the Cold Days red/pink shirts were a significant indicator of conception risk, (1, *N* = 108)  = 4.78, *p*  = .03 (Odds ratio  = 4.20); on the Warm Day shirt color did not significantly predict risk, 7% vs. 13%,(1, *N*  = 101)  = 0.81, *p*  = .37 (Odds ratio  = 0.55). Error bars indicate standard errors of the mean.

Next, we again classified women according to whether they reported wearing a red or pink shirt (*n  = *22) or an other-colored shirt (*n*  = 187). Using logistic regression, we tested whether Day moderated the effect of conception risk on shirt color. A significant interaction emerged, *B*  = 2.06, Wald (1)  = 4.49, *p*  = .03, indicating that, on the cold days, conception risk significantly predicted women’s shirt color, with 18% of high-risk women wearing red or pink compared to 5% of low-risk women,(1, *N*  = 108)  = 4.78, *p*  = .03 (Odds ratio  = 4.20). In contrast, on the warm day no significant effect emerged, 7% vs. 13%,(1, *N*  = 101)  = 0.81, *p*  = .37 (Odds ratio  = 0.55). Examining the likelihood that a woman’s shirt color choice predicted whether she was at high risk for conception, we found that, on the cold days, 75% of women wearing red or pink were at peak fertility, whereas on the warm day only 40% of women wearing red or pink were at peak fertility; see Figure 1. These results, when combined with those from our prior study [10], suggest that red or pink colored clothing is strongly associated with ovulation, and that the effect of conception risk on women’s tendency to wear red or pink is moderated by weather.

## Conclusions

The present studies demonstrate that: (a) the previously documented effect of conception risk on women’s tendency to wear red or pink is robust under certain, theoretically predictable circumstances, and (b) these circumstances are consistent with the theoretical expectation that women use red or pink dress as a way of increasing their apparent sexual attractiveness. That is, the finding that fertility is most predictive of women’s self-adornment in red or pink during colder weather is consistent with this theory because in warmer weather women can choose among numerous alternative means of communicating their sexual appeal through clothing choice.

These findings have several important implications. First, they provide confirmatory support for the prior finding that women at high conception risk are more likely to dress in red or pink. Although the present research suggests an important boundary condition on this effect, it also highlights the robustness of the effect when that condition is present; the effect size documented here on cold days is comparable to that observed in the similarly sized sample in our prior research ( [10]: Sample A, *n*  = 100). The large magnitude of this effect, now observed in three different samples of women participating during times of relatively cold weather, suggests that, under cold conditions, there is an objective, visible, behavioral display associated with ovulation.

Second, the finding that this behavioral display emerges reliably only in cold weather suggests that it is responsive to contextual factors, and is not a fully cross-generalizable response. In other words, unlike the highly visible physical signals of ovulation found in many other species (e.g., chimpanzees’ pinkish estrous swellings), human women’s tendency to wear red or pink when at peak fertility is not likely to be a fully biologically determined response; it is very unlikely that the hormones associated with ovulation directly increase women’s desire to wear red or pink. Rather, this behavioral tendency is likely to be a consequence of psychological changes that occur with ovulation, such as women’s increased desire to appear sexually attractive during this time [8]. Previous studies have provided compelling support for this tendency; women at high-conception risk show a significantly greater interest in wearing revealing clothing, dressing more fashionably, and purchasing sexier clothing to wear at a social gathering where they might meet men [8], [16], [23]. By demonstrating that the ovulation-linked tendency for women to wear red or pink is most apparent in contexts where there are few other acceptable means to visibly increase one’s sexual attractiveness, the present work provides evidence for a mediational pathway from biology (i.e., ovulation and associated hormones) to psychology (i.e., motivation to increase one’s sexual attractiveness) to behavior (increased tendency to wear red or pink when normative and comfort concerns prohibit wearing revealing or minimal clothing). Future studies are needed to directly test this full mediational model, most importantly by examining whether the currently observed effects are driven by an increased desire among high-conception-risk women to increase their sexual attractiveness, and a corresponding belief among these women that self-adorning in red or pink provides a viable means of doing so.

These findings also raise several other important questions which should be explored in future work. First, if weather is the key factor that determines whether red and pink dress are systematically linked to women’s fertility, studies are needed to examine exactly how cold it needs to be for the effect to emerge. Based on the present results, we can narrow the critical window to between 39°F and 57°F, and it may be that individual differences in sensitivity to cold prevent considerably greater specification. However, this is an interesting question for future work. In addition, there are likely to be other contexts that moderate the effect by increasing or decreasing women’s options for dressing in a sexually appealing yet socially normative manner. For example, building on the prior finding that women at high-conception risk wear more revealing clothing when at Austrian Discotheques [22], we might expect the presently observed red dress effect to be weaker in such circumstances, as well as at other social events geared toward facilitating dating and courtship, even during colder times. Future studies documenting such additional moderators will both help confirm the theoretical explanation for the present findings and indicate more precisely when and where red and pink dress can be viewed as a cue to fertility.

Second, the present findings are likely to be linked, in some manner, to men’s tendency to find red/pink attractive, which raises the question of *why* men are, cross-culturally, so attracted to these colors. Several researchers have suggested that this proclivity is the result of an adaptation originating in our non-human primate ancestors, resulting from the fact that, in some primate species (e.g., chimpanzees), females’ genitals acquire an extreme reddish or pinkish coloration during ovulation (i.e., estrous swellings), from increased vascularization [9], [28]. The visibility of these swellings would make it adaptive for males of these species to find redness in females attractive, and it is possible that a cognitive mechanism associating redness with attractiveness thus emerged in a shared non-human ancestor and was retained in humans. Importantly, this account presupposes that estrous swellings were present in at least some ancestor shared between humans and these other primates; it is thus challenged by evidence that exaggerated swellings emerged only after the chimpanzee genus diverged from the line that led to modern humans [29]. However, others have suggested that human ancestors may have displayed some form of considerably more subtle visual signs of estrus, including a slight reddening of the anogenital area [30], and that humans’ erect posture concealed this coloration such that, eventually, female fertility signaling through red or pink coloration became maladaptive due to the energy expended creating such displays (see [31]). Given these competing accounts, this is an issue that would benefit from future investigations.

Finally, one methodological limitation of the present research was our reliance on self-report rather than hormonal measures to assess conception risk. Although concerns have been raised regarding the reliability of the assessment method we used [26] (but see [27]), our invocation of a recently developed method to assess women’s confidence in their self-reported menses onset helps address this issue. By assessing women’s confidence in their self-reported menses onset date, this method takes into account women’s uncertainty on this issue, and thus ameliorates some concerns regarding the use of self-reported estimates. Nonetheless, future studies should seek to replicate and extend these findings using hormonal assessment techniques.

Regardless of these issues, which highlight important directions for future research, the present results indicate that under certain, theoretically predictable circumstances, female ovulation–long assumed to be hidden–is in fact associated with a distinct, objectively observable behavioral display.
